# Optimal pain management for radical prostatectomy surgery: what is the evidence?

**DOI:** 10.1186/s12871-015-0137-2

**Published:** 2015-11-04

**Authors:** Grish P. Joshi, Thomas Jaschinski, Francis Bonnet, Henrik Kehlet, on behalf of the PROSPECT collaboration

**Affiliations:** Department of Anesthesiology and Pain Management, University of Texas Southwestern Medical School, 5323 Harry Hines Blvd, Dallas, TX 75390-9068 USA; Institute for Research in Operative Medicine, Witten/Herdecke University, Cologne, Germany; Department d’ Anesthesie Reanimation, Hôpital Tenon, Assistance Publique Hôpitaux de Paris Université Pierre & Marie Curie, Paris, France; Section for Surgical Pathophysiology, Rigshospitalet, Copenhagen University, Copenhagen, Denmark

**Keywords:** Radical prostatectomy, Postoperative, Pain, Multimodal analgesia

## Abstract

**Background:**

Increase in the diagnosis of prostate cancer has increased the incidence of radical prostatectomy. However, the literature assessing pain therapy for this procedure has not been systematically evaluated. Thus, optimal pain therapy for patients undergoing radical prostatectomy remains controversial.

**Methods:**

Medline, Embase, and Cochrane Central Register of Controlled Trials were searched for studies assessing the effects of analgesic and anesthetic interventions on pain after radical prostatectomy. All searches were conducted in October 2012 and updated in June 2015.

**Results:**

Most treatments studied improved pain relief and/or reduced opioid requirements. However, there were significant differences in the study designs and the variables evaluated, precluding quantitative analysis and consensus recommendations.

**Conclusions:**

This systematic review reveals that there is a lack of evidence to develop an optimal pain management protocol in patients undergoing radical prostatectomy. Most studies assessed unimodal analgesic approaches rather than a multimodal technique. There is a need for more procedure-specific studies comparing pain and analgesic requirements for open and minimally invasive surgical procedures. Finally, while we wait for appropriate procedure specific evidence from publication of adequate studies assessing optimal pain management after radical prostatectomy, we propose a basic analgesic guideline.

## Background

Prostate cancer is the most common cancer in men, with more than 240.000 patients newly diagnosed per year in the United States alone [1]. Radical prostatectomy remains one of the key techniques to treat prostate cancer [2], and the incidence of surgery has risen with improved prostate-specific antigen screening programmes [3, 4].

Optimal pain management is known to influence postoperative recovery [5], but patients undergoing open radical prostatectomy typically experience moderate dynamic pain in the immediate postoperative days [6]. Robot-assisted and laparoscopic surgery may be associated with decreased pain levels as opposed to open surgery [6], but even here, abdominal and incisional pain are prominent sources of moderate dynamic pain scores [7, 8].

The literature assessing the efficacy of various analgesic drugs and techniques in patients undergoing radical prostatectomy has not been systematically evaluated. Consequently, optimal pain therapy for patients undergoing radical prostatectomy remains to be defined.

The aim of the present systematic review is to evaluate the available literature on the management of pain after radical prostatectomy. Postoperative pain outcomes (e.g., pain scores and supplemental analgesic requirements) are the primary focus, but other recovery outcomes, including adverse effects, are also assessed where reported, and the limitations of the data are reviewed. This systematic review will also be used to determine the knowledge gaps, which will guide future research. In addition, this review can serve as a starting point for developing recommendations for clinical decision-making in the management of pain after radical prostatectomy surgery.

## Methods

### Systematic literature search

Medline, Embase, and the Cochrane Central Register of Controlled Trials were searched for studies comparing analgesic and anesthetic interventions in patients undergoing radical prostatectomy according to the Preferred Reporting Items for Systematic reviews and Meta-Analyses (PRISMA) guidelines [9]. All searches were conducted in October 2012 without restriction to the publication date by using a combination of text words and data-base specific controlled terms related to prostatectomy, analgesia and pain assessment. We also manually retrieved publications referred in studies identified by our preceding search. The search was updated in June 2015.

### Study inclusion and selection

The selection process was performed in a two-step procedure. First, two reviewers selected studies independently by screening the titles and abstracts according to predefined inclusion criteria: randomized controlled trials (RCTs) published as full-text in English assessing analgesic, anesthetic and surgical techniques affecting postoperative pain in patients undergoing radical prostatectomy. In studies with mixed surgical procedures there had to be a defined prostatectomy subgroup. After retrieving potential relevant studies, full-texts were checked against the inclusion criteria once again. Any disagreements were resolved by consensus. In the case of insolvable discrepancies, a third reviewer was involved in the discussion.

### Quality assessment and outcome analysis

For the critical appraisal of included studies we used the Cochrane Collaboration’s tool for assessing the risk of bias [10]. The data extraction tables summarize pain scores, supplementary analgesic use and time to first analgesic requirement. It was assumed that the postoperative pain scores were assessed at rest, unless otherwise specified in the study report. Studies were stratified according to the regimen (analgesic, anesthetic and operative), mode of delivery (systematic or local) and class of agent. The assessment of the risk of bias and data extraction were conducted by one author and checked by a second author. Any disagreements were resolved by discussion or by consultation of a third reviewer. Quantitative meta-analyses were not performed, owing to the limited number of included studies with homogenous designs reporting similar outcome measures.

## Results

### Study selection process

**Fig. 1:**
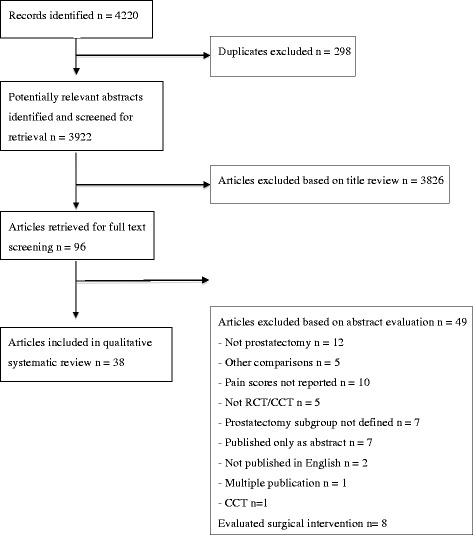
PRISMA diagram showing identification of included studies

In the search until October 2012, 38 studies met the inclusion criteria (Fig. 1), of which, an open approach was performed in 34 studies [11–43], a laparoscopic approach was performed in 1 study [44], and a robotic-assisted laparoscopic approach was performed in 3 studies [45–47]. Due to insufficient reporting the surgical approach was unclear in one study [48].

### Risk of bias in included studies

The quality of all included studies was moderate to poor and most studies had similar flaws (Table 1). For the qualitative analysis the trials were assigned to 2 broad groups: pharmacological techniques and anesthetic techniques. There were no studies that compared or utilized multimodal pain interventions.

**Table 1:** Methodological quality summary and level of evidence (LoE)

Study	Generation of allocation sequence	Allocation concealment	Blinding of participants and personnel	Blinding of outcome assessment	Incomplete outcome data	Selective outcome reporting	LoE
Allaire 1992 [11]	O	O	-	-	+	O	2
Andrieu 2009 [12]	O	O	-	-	O	-	2
Aribogan 2002 [13]	O	O	-	+	O	-	2
Bilgin 2011 [14]	+	O	+	+	O	-	1
Borazan 2010 [15]	+	O	+	O	O	O	1
Brown 2004 [16]	+	O	+	O	+	O	1
Chelly 2011 [17]	+	O	+	+	+	-	1
Fant 2011 [18]	+	O	+	O	+	O	1
Gaitini 1996 [19]	O	O	-	-	O	-	
Gottschalk 1998 [20]	O	O	+	+	O	O	1
Groudine 1998 [21]	+	O	+	+	+	O	1
Gupta 20 06 [22]	+	O	+	+	+	-	1
Habib 2008 [23]	O	O	+	+	+	O	1
Habib 20 09 [24]	+	+	+	+	+	-	1
Haythornthwaite 1998 [25]	O	O	-	+	O	O	
Heid 20 07 [26]	+	O	+	+	O	-	1
Hohwu 20 06 [27]	O	+	-	-	+	O	1
Hong 2011 [28]	+	O	+	+	+	O	1
Huang 2001 [29]	+	O	+	+	O	O	1
Katz 2004 [30]	+	+	+	+	+	O	1
Liu 1995 [31]	O	O	+	+	O	O	1
Mayson 2000 [32]	+	O	+	+	+	O	1
Mazaris 2008 [33]	O	O	_-_	_-_	O	O	2
Ormiston 1981 [34]	O	O	+	O	+	-	1
Salonia 20 06 [36]	+	O	-	-	O	O	2
Shir 1994 [37]	O	O	-	+	O	O	2
Snijdelaar 2004a [38]	+	O	+	O	+	O	1
Snijdelaar 2004b [39]	+	O	+	+	+	-	1
Tauzin-Fin 2006 [40]	+	O	+	+	O	O	1
Tauzin-Fin 2007 [41]	+	O	+	+	+	O	1
Tauzin-Fin 2009 [42]	+	O	+	+	+	O	1
Wu 2005 [43]	+	O	+	+	O	O	1
Lauwick 20 09 [44]	+	+	+	O	O	O	1
Hong 20 09 [45]	+	O	-	+	+	-	1
Lee 2011 [46]	O	O	-	+	O	O	
Lukasewycz 20 1 0 [47]	+	O	+	+	+	-	1
Larijani 2004 [48]	O	O	+	O	O	-	
Weinberg 2014 [49]	+	O	+	+	+	O	1
Fuller 2013 [50]	+	+	O	+	+	+	1
Dirkmann 2015 [55]	+	+	+	+	+	+	1
Nuri-Deniz 2013 [56]	+	O	-	+	+	+	1
Ozbek 2013 [57]	+	-	+	+	O	+	1
Elkassabany 2013 [59]	+	+	+	+	+	+	1
Kristensen 2013 [61]	+	+	+	+	+	+	1
Deniz 2012 [62]	+	-	-	O	+	O	1

‘+’ low risk of bias; ‘-’ high risk of bias; ‘O’ unclear risk of bias

### Pharmacological interventions

The trials assessing analgesic interventions were grouped into conventional analgesics (non-selective non-steroidal anti-inflammatory drugs (NSAIDs), cyclooxygenase (COX) 2-selective inhibitors, lidocaine, and opioids); adjunct drugs with analgesic activity (α2 agonists, α2δ ligands [gabapentin and pregabalin], muscarinic receptor antagonists and N-methyl-D-aspartic acid [NMDA] antagonists [magnesium and ketamine]) (Table 2); and regional anesthesia techniques generally showed that these pharmacological approaches were useful (Table 3). Four studies compared epidural analgesia with systemic analgesia, of which two showed a reduction in pain scores (Table 3). Two studies evaluated intrathecal opioids with or without clonidine (Table 3). Both showed improved pain relief, but increased frequency of pruritus was reported in one study.

**Table 2:** Summary of key results from included studies evaluating pharmacological interventions in patients undergoing radical prostatectomy

Reference	Intervention studied	Pain scores	Supplementary analgesia	Time to first analgesic request
Non-steroidal Anti-Inflammatory Drugs (NSAIDs)				
Mazaris 2008 [33]	lornoxicam versus paracetamol	↓at rest	NS	-
Ormiston 1981 [34]	aspirin versus tiaprofenic acid	NS at rest	-	-
Bilgin 2011 [14]	Diclofenac, IM versus placebo	↓↓ at rest	↓↓	-
Dirkmann 2015 [55]	Parecoxib versus placebo	↓↓	↓↓	-
Cyclo-oxygenase-2 Selective Inhibitors				
Huang 2001 [29]	rofecoxib versus placebo	NS at rest	NS	-
Chelly 2011 [17]	celecoxib versus placebo	↓at rest	↓	-
Lidocaine Infusion				
Groudine 1998 [21]	lidocaine versus placebo	↓↓ at rest	NS	-
Lauwick 2009 [44]	lidocaine versus placebo*	NS at rest	↓	-
Opioids				
Larijani 2004 [48]	morphine versus placebo	↓↓ at rest	-	NS
Gaitini 1996 [19]	buprenorphine versus morphine	NS at rest	-	-
Topical Administration				
Habib 2008 [24]	nicotine versus placebo	NS at rest and on coughing	↓↓	-
Habib 20 09 [23]	lidocaine versus placebo	↓↓ at rest and on coughing	NS	-
Analgesic Adjuncts				
a2 agonists				
Mayson 2000 [32]	clonidine versus placebo	NS at rest and on coughing	NS	-
Muscarinic receptor antagonists				
Tauzin-Fin 2007 [40]	oxybutynin versus placebo	↓↓ at rest	↓↓	-
Lukasewycz 20 1 0 [47]	belladonna and opium versus placebo*	↓ at rest NS on movement	NS	
N-methyl-D-aspartic acid (NMDA) antagonists				
Tauzin-Fin 2006 [40]	magnesium versus placebo	NS at rest	↓↓	NS
Katz 2004 [30]	ketamine versus placebo	NS at rest	NS	-
Snijdelaar 2004a [38]	ketamine versus placebo	↓↓ at rest NS on movement	↓↓	-
Snijdelaar 2004b [39]	amantadine versus placebo	NS at rest	↓↓	-
Melatonin				
Borazan 2010 [15]	melatonin versus placebo	↓↓ at rest	↓↓	-
Gabapentin				
Deniz 2012 [62]	Gabapentin versus placebo	↓↓ at rest for 2 h postop	NS	-

**Table 3:** Summary of key results from included studies evaluating local/regional analgesia techniques in patients undergoing radical prostatectomy (^a^ indicates laparoscopic or robotic approach)

Reference	Intervention studied	Pain scores	Supplementary analgesia	Time to first analgesic request
Epidural analgesia versus systemic analgesia				
Allaire 1992 [11]	Epidural fentanyl versus morphine	↓↓ at rest	-	-
Gupta 2004 [22]	Epidural ropivacaine, fentanyl and adrenaline plus placebo via IV-PCA versus epidural placebo and morphine via IV-PCA	↓↓ at rest and on coughing	-	-
Hohwü 2006 [27]	Epidural ropivacaine versus bupivacaine infiltration + oral oxycodone	NS	-	-
Liu 1995 [31]	Epidural hydromorphone versus hydromorphone via IV-PCA	NS at rest and on coughing	-	-
Perioperative epidural analgesia versus postoperative epidural analgesia				
Gottschalk 1998 [20]	Preemptive epidural fentanyl (4 μg/kg) versus preemptive epidural bupivacaine (5mg/ml) and postoperative morphine versus postoperative morphine and bupivacaine. All patients received postoperative epidural morphine (0.1mg/ml) and bupivacaine (0.5mg/mL)	↓↓ at rest in both preemptive groups	↓↓ in bupivacaine group only	-
Hong 2011 [28]	Epidural ropivacaine versus epidural ropivacaine (3mg/ml) plus sufentanil (1 μg/ml) versus epidural placebo	↓ at rest	↓↓	-
Components of epidural analgesia				
Aribogan 2003 [13]	Epidural combination of tramadol and bupivacaine versus tramadol only versus bupivacaine alone	↓↓ at rest	↓↓	-
Heid 2007 [26]	Epidural ropivacaine versus bupivacaine	NS at rest and on coughing	NS	-
Epidural analgesia versus local infiltration analgesia				
Fant 2011 [18]	Epidural ropivacaine and fentanyl versus ropivacaine via intra-abdominal catheter	↓↓ at rest and on coughing	↓↓	-
Intrathecal Opioids				
Andrieu 2009 [12]	Intrathecal morphine 4 μg/kg versus morphine 4 μg/kg plus clonidine 1 μg/kg versus placebo	↓↓ at rest and on movement in both treatment groups for 18 h. Clonidine extended duration by 6 h.	↓↓	↓
Brown 2004 [16]	Intrathecal morphine 0.2 mg and clonidine 75 μg	↓↓	↓↓	-
Nuri Deniz 2013 [56]	Intrathecal morphine 0.2 mg	↓↓	↓↓	-
Wound infiltration versus placebo				
Wu 2005 [43]	Subfascial bupivacaine versus placebo	NS at rest and on movement	NS	-
Kristensen 2013 [61]	subfascial bupivacaine versus placebo	NS	NS	-
Elkassabany 2013 [59]	TAP block versus placebo	↓↓	↓↓	-
Penile nerve block				
Weinberg 2014 [49]	Dorsal penile nerve block with bupivacaine vs. placebo^a^	NS	NS	-
Wound infiltration with magnesium				
Lee 2011 [46]	Magnesium under remifentanil-based anaesthesia versus placebo under remifentanil-based anaesthesia magnesium under remifentanil-based anaesthesia versus placebo under desflurane-based anaesthesia^a^	↓↓ on movement	↓↓	↓↓
		NS on movement	NS	NS
Tauzin-Fin 2009 [42]	Infiltration of ropivacaine plus magnesium versus infiltration of ropivacaine plus magnesium, IV	NS at rest	↓↓	↓↓

*NA* not analyzed, *NS* no significant difference between groups

- not reported

↓, decreased at a minority (50 % or less) of time points measured

↓↓, decreased at more than 50 % of time points measured

### Surgical techniques

Although a minimally invasive approach for radical prostatectomy has been rapidly adopted in clinical practice [3, 4], there are only 4 RCTs assessing pain management. Moreover, between October 2012 and June 2015, only 2 additional RCT have been published assessing pain control using a robotic approach [49, 50]. These studies focused on adjunct techniques (i.e., penile block to improve bladder catheter tolerance [49] and intravesical ropivacaine [50]) and both did not result in any improvement in pain control.

### Anesthetic techniques

Three studies investigating the use of regional anesthesia, including combined procedures with general anesthesia, showed a reduction of analgesic supplemental use with regional anesthesia (Table 4). However, the differences between groups with regard to pain scores were inconclusive. Two studies compared spinal anesthesia with general anesthesia. Patients receiving spinal anesthesia had significantly shorter durations of surgery, reduced blood loss and lower pain scores on the day of surgery than patients receiving general anesthesia.

**Table 4:** Summary of key results from included studies evaluating anesthetic interventions in patients undergoing radical prostatectomy (^a^ indicates laparoscopic or robotic approach)

Reference	Intervention studied	Pain scores	Supplementary analgesia	Time to first analgesic request
Shir 1994 [37]	RA versus GA	↓ at rest	↓↓	-
Haythornthwaite 1998 [25]	RA versus combined RA/ GA	NS	↓↓	-
Hong 2009 [45]	Combined RA/GA versus GA^a^	NS at rest, ↓↓ on coughing	↓↓	-
Salonia 2004 [35]	SA versus GA	↓ at rest	-	-
Salonia 20 06 [36]	SA versus GA	↓↓ at rest	-	-

*GA* general anesthesia, *RA* regional anesthesia, *SA* spinal anesthesia, *NS* no significant difference between groups

-, not reported

↓, decreased at a minority (50 % or less) of time points measured

↓↓, decreased at more than 50 % of time points measured

## Discussion

This systematic review reveals that there is a significant lack of evidence to develop an optimal pain management protocol in patients undergoing radical prostatectomy. Most studies evaluating pain management after radical prostatectomy surgery assessed unimodal analgesic approaches [11–48]. The optimal dose or timing of administration of analgesic agents could not always be determined. Although it is generally accepted that minimal access surgery for radical prostatectomy reduces postoperative pain, it is poorly studied.

Pain after laparoscopic/robotic prostatectomy is generally mild-to-moderate [7]. A recent observational, prospective cohort study that included a limited number of opioid-naïve patients reported that pain after robotic radical prostatectomy was adequately controlled primarily with NSAIDs and opioids [47]. Because opioids may delay recovery and increase the length of hospital stay [51], due to opioid-related adverse effects such as nausea, vomiting and prolonged postoperative ileus [52], non-opioid analgesics and/or regional analgesic techniques should be used as primary analgesics, and supplemented with opioids, only if necessary.

While we wait for appropriate procedure specific evidence for optimal pain management after minimally invasive radical prostatectomy, a basic analgesic technique, used in observational trials [7], could include a combination of acetaminophen (paracetamol) and NSAID or COX-2 selective inhibitor along with wound infiltration of the trocar sites [5]. The choice between a traditional NSAID and COX-2 selective inhibitors should depend upon assessment of individual patient risks. Non-selective NSAIDs can increase the potential risk of bleeding [53] in contrast to COX-2 selective inhibitors. However, a recent randomized, placebo-controlled, double-blind trial in patients undergoing open prostatectomy reported that while parecoxib reduced opioid use and opioid-related side effects, blood loss at 24 h after surgery was significantly higher in comparison to the placebo group, corresponding to a 1 g/dL difference in hemoglobin [54].

For patients undergoing open prostatectomy under spinal anesthesia, intrathecal morphine may be an appropriate alternative, assuming that proper precautions are taken for prevention of the morphine-related complications such as nausea and vomiting, pruritus, and respiratory depression. This is also supported by two recent studies reporting reduced intravenous opioid requirements after intrathecal morphine (150–200 μg), with a consequent decrease in the incidence of nausea and vomiting [55, 56]. However, there is a lack of data supporting superiority of epidural analgesia for this surgical procedure; two studies in this systematic review reported benefit from epidural analgesia [11, 22], while two studies found no benefit of epidural analgesia over systemic analgesia [27, 31]. A recent study published after the completion of the systematic review reported that epidural analgesia increased by one day, the length of hospital stay and recommended its avoidance [57].

Two recent studies published after the deadline for inclusion in this systematic review, report controversial results concerning the analgesic effect of the transversus abdominis plane (TAP) blocks included in multimodal protocols [58, 59]. One placebo-controlled study published after the deadline of this systematic review reported that postoperative local anesthetic infusion via a subfascially placed wound catheter did not improve pain relief when combined with basic analgesic regimen consisting of acetaminophen and NSAID with opioid used as rescue [60].

The limitations of this systematic review stem from the limitations of the included studies: particularly the inadequate study design (e.g., lack of double-blinding or explicit randomization) and lack of use of simple non-opioid analgesics when comparing more invasive techniques and a failure to evaluate all the potentially relevant analgesic agents and techniques for radical prostatectomy (especially infiltration techniques).

Thus, this review has identified several areas for future research when current data are insufficient or conflicting. There is a need for clinical trials evaluating multimodal analgesia techniques that would include combinations of paracetamol and NSAID/COX-2 selective inhibitor, and regional anesthetic techniques, with oral opioids administered only as rescue postoperatively. Future studies also need to evaluate the benefit to risk of continuous local anesthetic wound infusion and TAP blocks combined with multimodal analgesia. Also, large randomized clinical trials are necessary to assess the efficacy as well as optimal dose and duration of lidocaine intravenous infusion, ketamine and gabapentinoids. A study published after the deadline reported that a single preoperative dose (900 mg) of gabapentin reduced pain scores but not opioid requirements [61].

Future trials should include multimodal enhanced rehabilitation protocols (fast track or enhanced recovery programs) as an integral part of the study design [62]. This will allow us to differentiate the effects of the analgesic interventions on perioperative outcome from those of the enhanced recovery programs that are becoming the standard of care. Also, there is a need for more procedure-specific studies comparing pain and analgesic requirements between open and minimal access (laparoscopic and robotic) surgical procedures.

## Conclusions

This systematic review reveals that there is a lack of evidence to develop an optimal pain management protocol in patients undergoing radical prostatectomy. Most studies assessed unimodal analgesic approaches rather than a multimodal technique. There is a need for more procedure-specific studies comparing pain and analgesic requirements for open and minimally invasive surgical procedures. Finally, while we wait for appropriate procedure specific evidence from publication of adequate studies assessing optimal pain management after radical prostatectomy, we propose a basic analgesic guideline.

## Abbreviations

PRISMA: Preferred reporting items for systematic reviews and meta-analyses
RCTs: Randomized controlled trials
NSAIDs: Non-steroidal anti-inflammatory drugs
COX-2: Cyclooxygenase-2
NMDA: N-methyl-D-aspartic acid
TAP: Transversus abdominis plane
